# Artificial Intelligence Chatbots in Pediatric Emergencies: A Reliable Lifeline or a Risk?

**DOI:** 10.7759/cureus.89234

**Published:** 2025-08-01

**Authors:** Seerat Kular, Vikas Kumar

**Affiliations:** 1 Medicine, All India Institute of Medical Sciences, Bathinda, Bathinda, IND; 2 Pharmacology, All India Institute of Medical Sciences, Bathinda, Bathinda, IND

**Keywords:** american heart association, artificial intelligence, basic life support, chatgpt, pediatric emergency

## Abstract

Introduction

Artificial intelligence (AI) chatbots have rapidly gained popularity for disseminating health information, especially with the growth of digital medicine in recent times. Recent studies have shown that Chat Generative Pre-Trained Transformer (ChatGPT; OpenAI, San Francisco, CA), a widely used AI chatbot, has at times surpassed emergency department physicians in diagnostic accuracy and has passed basic life support (BLS) exams, underscoring its potential for emergency use. Parents are a key demographic for online health information, frequently turning to these chatbots for urgent guidance during child-related emergencies, such as choking incidents. While research has extensively examined AI chatbots' effectiveness in delivering adult BLS guidelines, their accuracy and reliability in providing pediatric BLS guidance aligned with American Heart Association (AHA) standards remain underexplored. This gap raises concerns about the safety and appropriateness of relying on AI chatbots for guidance in pediatric emergencies. In light of this, we hoped that comparing the performance of two ChatGPT versions, ChatGPT-4o and ChatGPT-4o mini, against established pediatric protocols by AHA could help optimize their integration into emergency response frameworks, providing parents with reliable assistance in critical situations. This analysis can pinpoint improvements for real-world integration, ensuring trustworthy assistance in critical situations.

Methodology

A prospective comparative content analysis was conducted between responses from ChatGPT (version 4o and its mini version) against the 2020 AHA Guidelines for Cardiopulmonary Resuscitation and Emergency Cardiovascular Care. The analysis focused on pediatric BLS, utilizing 13 broad questions designed to cover all key components, including fundamental concepts like the pediatric chain of survival and specific emergencies such as choking. Responses were evaluated for completeness and conformity to AHA guidelines. Completeness of the responses was analyzed as ‘Completely Addressed’, ‘Partially Addressed’, or ‘Not Addressed’, with partial responses further classified as ‘Superficial’, ‘Inaccurate’, or ‘Hallucination’. Conformity of responses to AHA 2020 guidelines was similarly analyzed and classified. Assessment of reliability was performed using Cronbach's alpha. Cohen’s kappa was used to check for interrater agreement between responses generated from two separate devices for the same set of questions.

Results

Content analysis of ChatGPT responses revealed that only 9.61% were fully addressed, and just 5.77% fully conformed to the AHA 2020 pediatric BLS guidelines. A majority of the responses (61.54%) were partially addressed and lacked depth, while 59.61% conformed only partially and superficially to the guidelines. Additionally, 5.77% of the queries were not addressed at all. ChatGPT-4o responses were generally more detailed and comprehensive compared to those from ChatGPT-4o mini. Inter-rater agreement ranged from slight to substantial between the two users.

Conclusions

While chatbots may assist with basic guidance, they lack the accuracy, depth, and hands-on instruction crucial for life-saving procedures. Misinterpretation or incomplete information from chatbots could lead to critical errors in emergencies. Hence, widespread BLS training remains essential for ensuring individuals have the practical skills and precise knowledge needed to respond effectively in real-life situations.

## Introduction

Artificial intelligence (AI) is revolutionizing healthcare by enhancing diagnostics, personalizing treatment plans, and improving patient outcomes through data-driven insights and automation. Among these advancements, AI chatbots are being utilized to provide automated responses, aid in preliminary assessments, and facilitate patient interactions within healthcare systems [1]. Before the advent of AI, rule-based chatbots were popular, and they had been based on a fixed, predefined set of rules and answered only a limited set of questions [2]. AI chatbots represent a more advanced type of chatbot that uses machine learning and natural language processing technologies. This enables AI chatbots to generate dynamic and human-like responses to a wide range of queries. They also learn and adapt over time, improving their ability to respond accurately. One such AI chatbot that attracts millions of users is Chat Generative Pre-trained Transformer (ChatGPT), which was developed by OpenAI in 2022 [3]. ChatGPT is trained on a vast dataset of books, articles, websites, and other publicly available content. When given a prompt, it uses its acquired knowledge to generate a response.

While the initial ChatGPT model is pre-trained on large-scale data, it can be fine-tuned to perform specific tasks like customer support. With the public increasingly turning to ChatGPT for health information in the same way as they do for other topics, concerns around the accuracy of its responses become crucial. Healthcare professionals thus have a critical role, as they can evaluate the content of such chatbots through research, educate the public on limitations based on findings, and advocate for their safe use. Douglas et al. conducted a study in 2023 to check the accuracy and completeness of answers provided by ChatGPT to a set of medical questionnaires. The study highlighted multiple instances where the chatbot came to a totally mistaken conclusion, which was delivered authoritatively and convincingly [4]. To further test the knowledge of ChatGPT in critical situations, a comparative analysis was performed between the European Resuscitation Council guidelines 2021 and ChatGPT 3.5 and 4. It was found that ChatGPT failed to address two-thirds of the key messages of the guidelines [5].

Despite its potential pitfalls in terms of the chance of incorrect information, ChatGPT continues to astonish healthcare professionals and the public alike. In a study comparing the emergency department (ED) physician diagnosis against ChatGPT’s diagnosis, it was found that ChatGPT surpassed the ED physicians in terms of diagnostic accuracy. It was found that the potential of ChatGPT lies in its capacity to generate a range of differential diagnoses, encompassing even rare diseases, making AI chatbots an important ally in a time-critical environment [6]. ChatGPT has recently drawn a lot of media attention with a mother detecting the cause of her son’s illness after many doctors could not diagnose it for years. ChatGPT has over 200 million weekly users today, highlighting the need for medical research to focus on validating health information available on it.

Parents are a key demographic for online health information, frequently turning to these chatbots for urgent guidance during child-related emergencies, such as choking incidents. Also, while substantial research has focused on adult basic life support (BLS) protocols, there is limited evaluation of how well these systems deliver pediatric BLS guidance, especially when measured against American Heart Association (AHA) standards. In this study, we aim to evaluate the performance of ChatGPT-4o, the most advanced and reliable model currently available under the ChatGPT framework [2], in comparison to its free counterpart, ChatGPT-4o mini. Both models are actively being utilized in the latest research [7]. The responses of both these models were compared against the benchmark of the latest pediatric BLS guidelines of the AHA, which are the AHA 2020 Guidelines for Cardiopulmonary Resuscitation and Emergency Cardiovascular Care [8].

## Materials and methods

Study design

We conducted a prospective comparative content analysis to evaluate the responses generated by ChatGPT versions 4o and 4omini to pediatric BLS queries, comparing them against the AHA 2020 Pediatric BLS guidelines.

Prompt development

Thirteen broad questions were developed by two BLS-trained investigators to comprehensively cover all critical aspects of the AHA 2020 pediatric BLS guidelines (Table 1).

**Table 1:** Questions provided as prompts to chatGPT BLS: basic life support; CPR: cardiopulmonary resuscitation

Question number	Question
1	What are the pediatric chain of survival guidelines for in-hospital settings?
2	What are the pediatric chain of survival guidelines for out-of-hospital settings?
3	How should CPR be initiated for infants and children according to pediatric BLS guidelines?
4	What are the key components of high-quality CPR for infants and children?
5	What is the proper technique for performing CPR on infants and children?
6	What are the recommended support surfaces for performing CPR on infants and children?
7	How do you properly open the airway for infants and children during pediatric BLS?
8	What is the pediatric BLS algorithm for healthcare providers in a single-rescuer scenario?
9	What is the pediatric BLS algorithm for healthcare providers when two or more rescuers are present?
10	What is the complete pediatric cardiac arrest algorithm?
11	What is the recommended treatment for inadequate breathing with a pulse in infants and children?
12	How should foreign body airway obstruction (choking) in infants and children be managed according to BLS guidelines?
13	What are the most recent updates to the pediatric BLS guidelines published by the American Heart Association?

AI response generation

The questions were given as prompts to ChatGPT versions 4o and 4o mini on two different devices. This yielded four sets of responses for the 13 questions: 4o1 (device 1), 4o mini1 (device 1), 4o2 (device 2), and 4o mini2 (device 2)

Content analysis

The responses generated by ChatGPT were evaluated by two BLS-trained investigators by comparing them to AHA 2020 guidelines [8]. To minimize bias, the raters were blinded to the AI model version and device used to generate each response. The method for the content analysis was adopted from a similar study in the past [5], which assessed AI-generated medical content using a structured scoring system that assessed completeness and conformity.

Two parameters were used: completeness and conformity. 

Completeness 

Completeness of responses meant whether the responses fully addressed the query. It was scored on a scale of 0 to 4 as follows:

Score 4: Fully addressed - responses that comprehensively covered all aspects of the query, leaving no omissions.

Score 3: Partially addressed-superficial - superficial meant that answers by ChatGPT covered some aspects of the query but failed to meet the required depth. Example: chest compressions are mentioned, but depth is not.

Score 2: Partially addressed-inaccurate - inaccurate meant that answers included details that contradicted the AHA guidelines. Example: incorrect depth of compressions mentioned.

Score 1: Partially addressed-hallucination - hallucination meant that the answers contained information that appeared credible but lacked evidence from the AHA guidelines. Example: talking about consent and team support in the BLS protocol.

Score 0: Not addressed - responses that were either completely irrelevant or failed to address the query.

Conformity

Conformity assessed whether the content adhered to the technical recommendations and content outlined in the AHA guidelines. It was further graded similarly from 0 to 4.

Reliability assessment

Inter-rater agreement was measured using Cohen’s kappa, while the reliability of the evaluation framework was assessed using Cronbach’s alpha.

Statistical analysis

Descriptive statistics were used to summarize results. Categorical variables were presented as frequencies and percentages. Cohen’s kappa was calculated to assess inter-rater agreement. Cronbach’s alpha was used to evaluate the reliability of the scoring tool. All statistical analysis was performed using SPSS Statistics version 29 (IBM Corp., Armonk, NY). A p-value <0.05 was considered statistically significant

Ethical considerations

This study did not involve human participants or identifiable personal data. It exclusively involved the analysis of AI-generated content.

## Results

**Figure 1:**
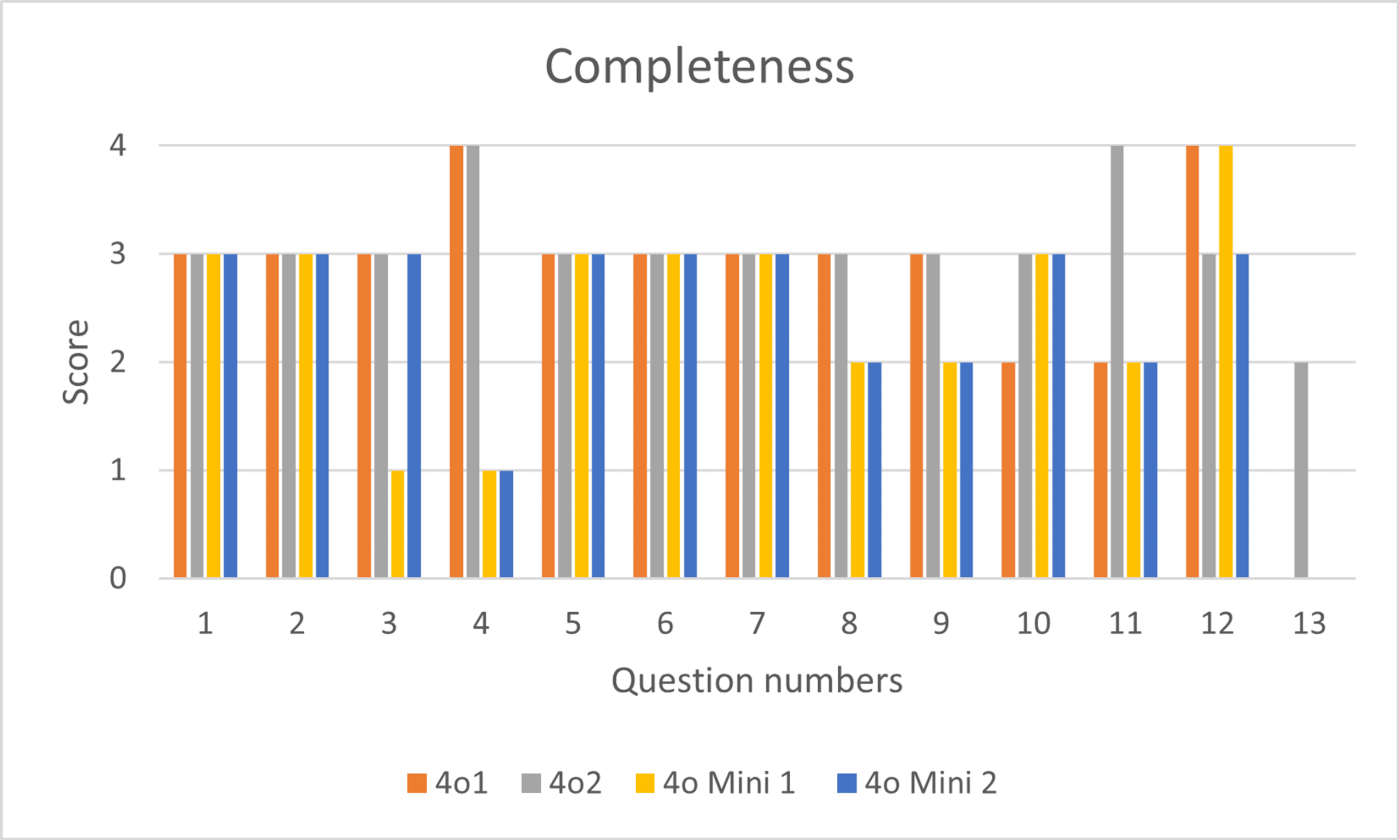
Completeness of responses of chatGPT 4o and 4o mini in both devices (1 and 2) as per AHA 2020 guidelines AHA: American Health Association; ChatGPT: Chat Generative Pre-trained Transformer

**Figure 2:**
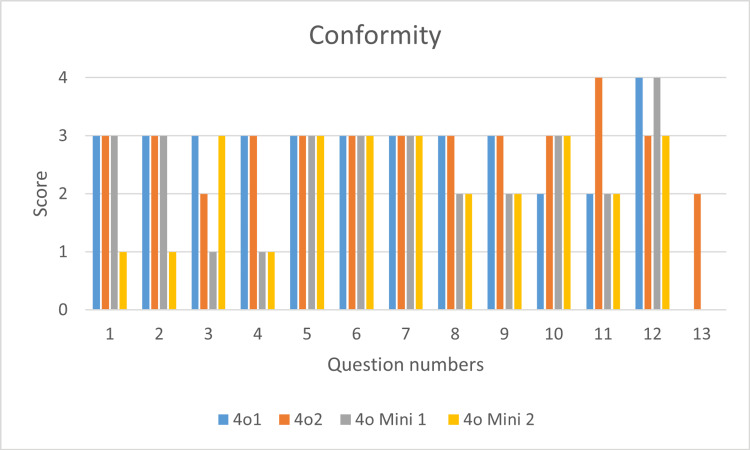
Conformity of responses of chatGPT 4o and 4o mini in both devices (1 and 2) as per AHA 2020 guidelines AHA: American Health Association; ChatGPT: Chat Generative Pre-trained Transformer

In response to the prompts of 13 questions framed based on the key points of the AHA guidelines for pediatric BLS, the statements of ChatGPT (version 4o and version 4o mini) were obtained and critically assessed for completeness (Figure 1) and conformity (Figure 2).

**Figure 3:**
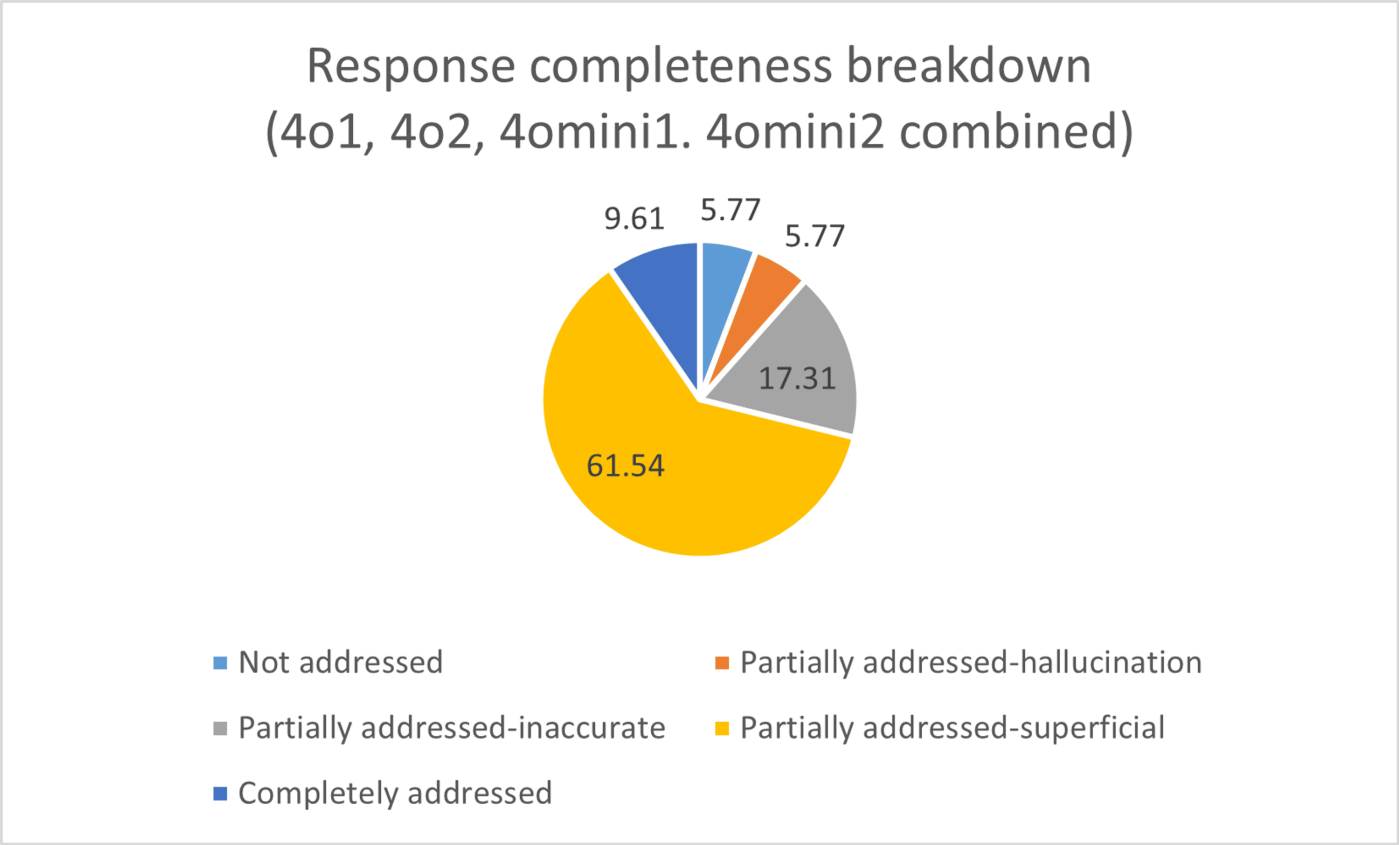
Response completeness breakdown (4o1, 4o2, 4o mini1, 4o mini2 combined)

**Figure 4:**
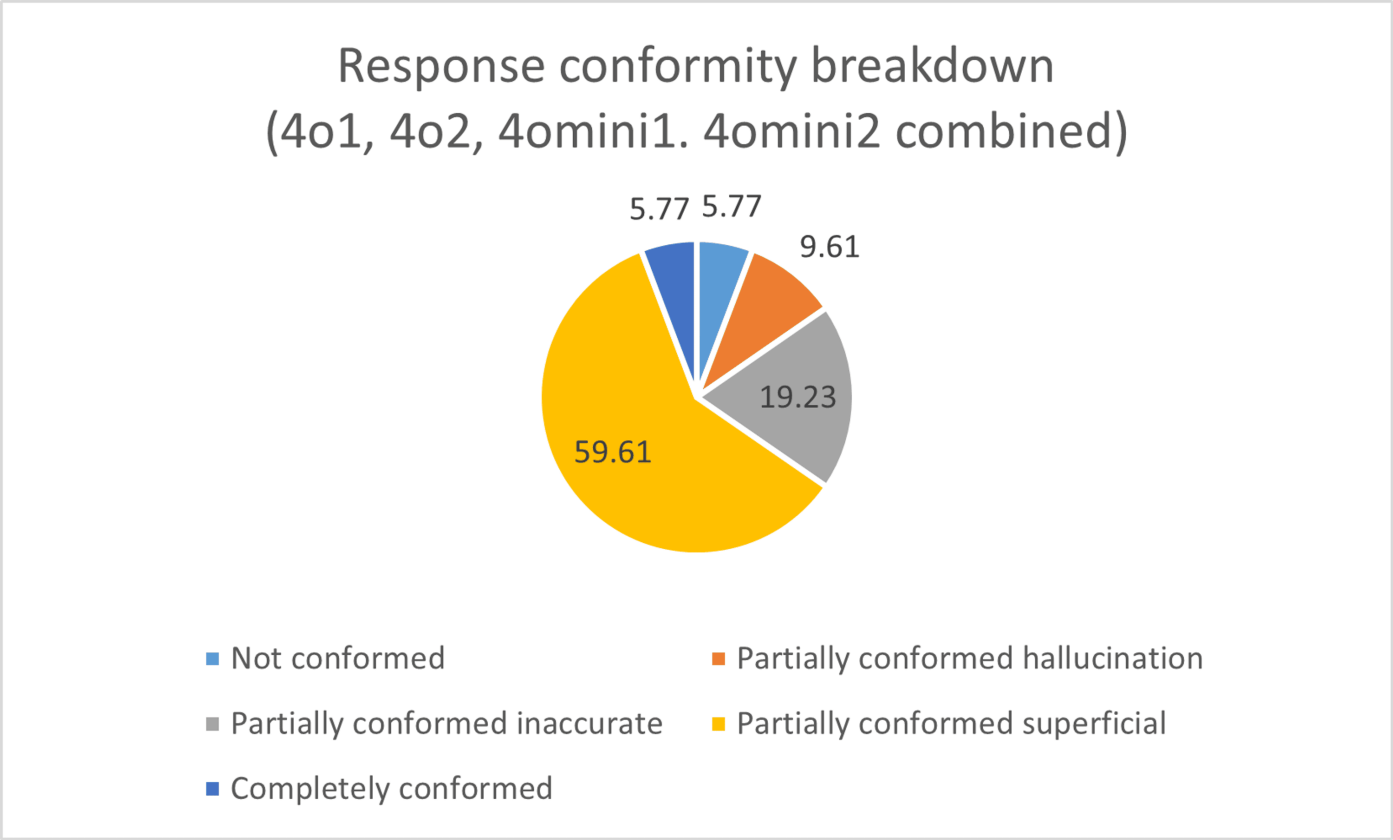
Response conformity breakdown (4o1, 4o2, 4o mini1, 4o mini2 combined)

Further content analysis revealed that only 9.61% of responses were fully addressed (Figure 3), and only 5.77% fully conformed to the AHA 2020 guidelines (Figure 4). The majority (61.54%) of responses were partially addressed and superficial, and 59.61% of responses conformed partially and were superficial when compared to the AHA 2020 guidelines; 5.77% of queries were not addressed. The proportion of hallucinated content was higher for ChatGPT 4o mini (11.5% of responses) as compared to ChatGPT 4o (3.8% of responses). Also, the responses given by ChatGPT 4o were more detailed and comprehensive than those given by 4o mini.

Completeness of responses

The majority of responses were only partially addressed and lacked depth. Out of the 13 queries evaluated (Table 2), only two were answered completely, and that too only by the GPT-4o model. These two queries pertained to the essential components of high-quality CPR and the protocol for managing choking. However, even within the GPT-4o model, inconsistencies were observed. For instance, the response from the second user regarding the choking protocol failed to include critical guidance, such as avoiding blind finger sweeps, making the answer superficial. In another instance, the GPT-4o mini model’s response on high-quality CPR focused on general aspects like teamwork, communication, and environmental safety, rather than the specific evidence-based components outlined by the AHA 2020, such as correct chest compression depth and rate.

**Table 2:** Completeness of responses

Responses	4o1	4o mini1	4o2	4o mini2
Not addressed	1/13	1/13	0/13	1/13
Partially addressed-hallucination	0/13	2/13	0/13	1/13
Partially addressed-inaccurate	2/13	3/13	1/13	3/13
Partially addressed-superficial	8/13	6/13	10/13	8/13
Completely addressed	2/13	1/13	2/13	0/13

Furthermore, the responses did not incorporate the most recent guideline updates. When prompted about the latest changes, the models largely reiterated existing BLS protocols without highlighting a significant 2020 revision concerning infants and children. This update specifies that if a pulse is present but respiratory effort is absent or inadequate, one breath should be delivered every two to three seconds, in contrast to the previous recommendation of one breath every three to five seconds. Notably, this critical update was omitted in most responses and was correctly mentioned only once by the GPT-4o model in its reply to the second user.

Conformity of the responses

The only fully conforming response was observed for the choking protocol (Q12) (Table 3). Several inaccuracies were noted in other responses (Table 4). For instance, lay rescuers were advised to check for a pulse before initiating CPR, whereas AHA guidelines recommend starting CPR immediately without pulse checks. The pediatric BLS algorithm for two or more rescuers was incorrectly stated as 30:2 compressions-to-breaths instead of the correct 15:2 ratio. Errors were also seen in the pediatric epinephrine dose and the frequency of rescue breaths for children. Partially conforming responses often included hallucinated content - such as references to teamwork, communication, education, and quality improvement - when specific technical details were expected (Table 5). These additions, though sounding plausible, were not aligned with the AHA 2020 guidelines and appeared to be fabricated to extend the response length. Notably, such hallucinations were present only in the 4o mini version; the 4o version did not exhibit these issues.

**Table 3:** Conformity of responses

Responses	4o1	4o2	4o mini1	4o mini2
Not conformed	1/13	0/13	1/13	1/13
Partially conformed-hallucination	0/13	0/13	2/13	3/13
Partially conformed-inaccurate	2/13	2/13	3/13	3/13
Partially conformed-superficial	9/13	10/13	6/13	6/13
Completely conformed	1/13	1/13	1/13	0/13

**Table 4:** Inaccurate responses by ChatGPT according to AHA 2020 AHA: American Health Association; BLS: basic life support; ChatGPT: Chat Generative Pre-trained Transformer; CPR: cardiopulmonary resuscitation; IO: intraosseous; IV: intravenous

Question number	Key point asked to ChatGPT	Version and user	Inaccurate information	Information according to AHA 2020
3	Initiation of CPR	4o2	Check breathing and pulse before starting CPR	Lay rescuers should begin CPR for any unresponsive victim, not breathing normally, and not have signs of life; do not check for a pulse
9	Pediatric BLS algorithm for 2 or more rescuers	4o mini1, 4o mini2	Cycle of 30 compressions followed by 2 breaths	When 2nd rescuer arrives, perform cycles of 15 compressions and 2 breaths
10	Pediatric cardiac arrest algorithm	4o1	Epinephrine: 1 mg/kg of 1:10000 IV/IO every 3-5 minutes during resuscitation	Epinephrine IV/IO dose: 0.01 mg/kg, max dose mg/kg; repeat every 3-5 minutes
11	Inadequate breathing with a pulse in infants and children	4o1, 4o mini1, 4o mini2	Rescue breaths - infants: 1 breath every 2-3 seconds; children: 1 breath every 3-5 seconds	For both infants and children: 1 breath every 2-3 seconds

**Table 5:** Hallucinated responses given by ChatGPT ChatGPT: Chat Generative Pre-trained Transformer; CPR: cardiopulmonary resuscitation

Question number	Key point asked to ChatGPT	Version and user	Hallucination
1	Pediatric chain of survival in the hospital	4o mini2	Continuous training and education
2	Pediatric chain of survival out of the hospital	4o mini2	Continuous training and quality improvement
3	Initiation of CPR	4o mini1	Bystanders may perform compression-only CPR if untrained
4	Components of high-quality CPR	4o mini1, 4o mini2	Teamwork and communication

Overall, most responses were only partially accurate and contained superficial errors. They lacked the depth and precision necessary for real-world application. Common omissions were noted across both versions in these partially addressed responses (Table 6).

**Table 6:** Areas receiving superficial responses by ChatGPT BLS: basic life support; ChatGPT: Chat Generative Pre-trained Transformer; CPR: cardiopulmonary resuscitation

	Key point asked to ChatGPT	The point that was ignored, rendering the response superficial
1, 2	Pediatric chain of survival in and out of the hospital	Recovery
3	CPR initiation	Initiate CPR with compressions airway breathing over airway breathing compression
4	Components of high-quality CPR	Once a child reaches puberty, use adult compression depth of at least 5 cm
5	CPR technique	For infants, if unable to achieve the guideline recommendation, it may be reasonable to use the heel of one hand
6	Support surfaces	During in-hospital cardiac arrest, when available, activate beds CPR mode to increase mattress stiffness
7	Opening the airway	For a trauma patient with suspected cervical spinal injury, if the jaw thrust does not open the airway, use the head tilt chin lift maneuver
8,9	Pediatric BLS algorithm	Case of normal breathing, pulse felt and no normal breath pulse felt not mentioned

Reliability

Cronbach’s alpha scores were 0.767 for completeness, indicating acceptable responses, and 0.818 for conformity, indicating good conformity. Rater’s agreement ranged from slight to substantial agreement (Table 7).

**Table 7:** Reliability

Observed data	Weighted kappa	Interpretation
Completeness: 4o1 vs. 4o2	0.260 (between 0.21 and 0.40)	Fair agreement
Completeness: 4o mini1 vs. 4o mini2	0.775 (between 0.61 and 0.80)	Substantial agreement
Coformity: 4o1 vs. 4o2	0.117 (between 0.00 and 0.20)	Slight agreement
Coformity: 4o mini1 vs. 4o mini2	0.536 (between 0.41 and 0.60)	Moderate agreement

## Discussion

The findings of our study align with concerns raised in prior research. One study evaluating ChatGPT’s responses to the European Resuscitation Guidelines found that 76.74% of key messages were not addressed at all [5]. Interestingly, that study reported a higher overall conformity rate (84%), likely due to the limited scope of guidelines tested. Across both studies, the most frequent causes of non-conformity were superficial responses and factual inaccuracies, underscoring the tendency of large language models to produce plausible yet clinically inadequate outputs. This issue was similarly observed in another study, where ChatGPT demonstrated 94% diagnostic accuracy for pediatric emergency conditions but advised calling emergency services in only 54% of cases and provided correct first aid instructions in just 45%. Alarmingly, incorrect advanced life support techniques were recommended in 13.6% of the scenarios [9].

Additional findings reinforce this concern. One study noted that while ChatGPT performed relatively well in adult BLS, its performance significantly declined in pediatric and infant scenarios, consistent with the results of our evaluation [10]. Although another investigation reported that ChatGPT outperformed emergency physicians in diagnostic accuracy [6], its clinical utility should be viewed with caution. While ChatGPT may be helpful as a diagnostic adjunct, particularly for ruling out rare conditions, its unreliability in providing accurate emergency protocol guidance, especially pediatric BLS, limits its application in real-time clinical settings.

Our inter-rater reliability analysis showed fair agreement for ChatGPT-4o and slight agreement for conformity scores. In contrast, a previous study comparing ChatGPT-3.5 and 4 found moderate reliability for completeness and fair to substantial reliability for conformity [5]; however, it did not assess inter-user variability, a key feature of our study design. Consistent with earlier literature [7,11], ChatGPT-4o outperformed the 4o mini version, providing more comprehensive and less hallucinated responses. Nonetheless, both models exhibited important limitations. Many responses lacked sufficient technical detail and contained inaccurate technical details, which were frequently embedded in otherwise correct-seeming answers. Such errors, particularly in high-stakes contexts, can pose serious risks in clinical decision-making.

The clinical consequences of these inaccuracies depend heavily on the end-user. Trained healthcare professionals may be able to detect and correct such errors. However, laypersons or first responders may follow incorrect advice, such as improper CPR technique or timing, potentially compromising patient outcomes. While broadly correct, AI-generated responses may still enhance awareness and promote action in otherwise passive bystanders. For clinical use, especially in critical areas like resuscitation, AI chatbots must evolve into expert-supervised or rule-based systems to ensure precision, reliability, and safety [12]. At the same time, widespread public training in BLS by certified organizations should continue to be a priority.

Limitations

Several limitations of this study should be acknowledged. Firstly, this evaluation was based on 13 broad questions derived from the AHA 2020 pediatric BLS guidelines. Although comprehensive, these questions could have been further subclassified to enable a more granular assessment of AI accuracy. Second, only two AI models (ChatGPT-4o and ChatGPT-4o mini) were tested. Inclusion of additional models such as Google Gemini, Bard, Android AI, or other emerging platforms would provide a more robust comparison. Third, our analysis focused solely on AHA guidelines. Future studies incorporating protocols from the European Resuscitation Council or national standards (e.g., Indian pediatric resuscitation protocols) would improve generalizability. Lastly, although evaluations were conducted by two trained reviewers, expanding the panel size and diversity could enhance objectivity and scoring consistency.

## Conclusions

While ChatGPT does address BLS guidelines, its responses are frequently superficial, lacking the depth and specificity required for clinical applicability. Among the evaluated models, ChatGPT-4o mini was found to be particularly unreliable, often generating hallucinated content, i.e., information that appears plausible but is not grounded in the AHA 2020 guidelines. In its current form, ChatGPT cannot be considered a dependable source for clinical decision-making or training in pediatric BLS due to recurring issues of inaccuracy, insufficient detail, and content hallucination. Nevertheless, these findings underscore its potential: with further development, including integration of rule-based logic, real-time guideline updates, and medical validation, ChatGPT could be refined into a reliable, guideline-conforming BLS chatbot that supports healthcare education and clinical preparedness in alignment with AHA standards.
